# Whole genome analysis, detoxification of ochratoxin a and physiological characterization of a novel *Bacillus velezensis* MM35 isolated from soil

**DOI:** 10.3389/fmicb.2024.1497860

**Published:** 2024-12-18

**Authors:** Fengru Xu, Mengmeng Tang, Zhihao Yang, Chengshui Liao, Zuhua Yu, Rongxian Guo, Ke Shang, Songbiao Chen, Ke Yang, Jing Li, Ke Ding, Yanyan Jia

**Affiliations:** ^1^College of Animal Science and Technology/Laboratory of Functional Microbiology and Animal Health, Henan University of Science and Technology, Luoyang, China; ^2^Luoyang Key Laboratory of Live Carrier Biomaterial and Animal Disease Prevention and Control, Luoyang, China; ^3^The Key Lab of Animal Disease and Public Health, Henan University of Science and Technology, Luoyang, China

**Keywords:** ochratoxin A, biodegradation, *Bacillus velezensis*, whole genome analysis, physiological characterization

## Abstract

Ochratoxin A (OTA) is a significant global contaminant that poses severe challenges to food safety and public health. This study aims to isolate the OTA-degrated probiotics and evaluate genetic and biological characteristic. Here, The degradation rate of a new strain named *Bacillus velezensis* MM35 isolated from soil was the highest (87.10% within 48 h), and its culture supernatant was the main component of OTA degradation (63.95%) by high performance liquid chromatography. Further investigation revealed that the extracellular enzyme that degrades OTA in the culture supernatant of MM35 may be a small molecule enzyme with certain heat resistance. Genome-wide analysis showed that MM35 contains a cluster of carboxypeptidases encoding OTA-degrading potential, and had good metabolic and catalytic synthesis ability, and strong application potential in the synthesis and degradation of carbohydrates and proteins. A variety of secondary metabolites with antibacterial properties, such as non-ribosomal peptide synthetase and terpenoids, were identified in its metabolites. Consistent with the predicted results, MM35 showed various enzyme production characteristics such as cellulase and xylanase. Furthermore, MM35 could inhibit the growth of a variety of pathogenic bacteria, and showed high co-aggregation ability to *Escherichia coli* and *Salmonella typhimurium*. In addition, MM35 has certain tolerance to harsh environments such as strong acid, bile salt, and high temperature. Additionally, the adhesion rate of MM35 was 5.4%, and the invasion rate was 2.1% in IPEC-J2 cells. In summary, the data suggest MM35 isolated strain has high OTA degradation efficiency, antibacterial activity and intestinal colonization, which provided a new way for the treatment of OTA contamination in food and feed industries.

## Introduction

Ochratoxins (OTs) are one of the five major mycotoxins of widespread concern currently. OTs are divided into four categories: A, B, C, and D. They are primarily produced by certain strains of Aspergillus, notably *Aspergillus ochraceus* and *Aspergillus niger*, as well as by some strains of *Penicillium*. These mycotoxins pose significant health risks to humans and animals, including nephrotoxic, immunotoxic, and carcinogenic effects, thereby necessitating ongoing monitoring and control in food and feed products. Among the various ochratoxins, ochratoxin A (OTA) is the most closely associated with human health. It exhibits the highest level of toxicity, is the most widely distributed, and presents the most severe contamination issues in agricultural products. OTA’s presence in food and feed raises significant concerns due to its strong toxic effect, making it a critical target for food safety and public health interventions (Liu et al., 2022; Qing et al., 2022; Khoi et al., 2021). The kidneys are the main target organ of OTA. OTA’s nephrotoxic effects are well-documented, and its accumulation in renal tissues can lead to chronic kidney disease and, in severe cases, kidney failure. After animals consume contaminated feed, their production performance can be adversely affected (Li et al., 2021). During this period, OTA can accumulate in animal tissues, milk, eggs, and other animal-derived foods, thereby posing a significant threat to human health. Studies have shown that exposure to OTA can impair various human functions and even lead to severe kidney-related conditions such as acute renal failure and chronic interstitial nephritis. In 2017, the International Agency for Research on Cancer (IARC) of the World Health Organization classified OTA as a Group 2B carcinogen, indicating that it is possibly carcinogenic to humans (Malir et al., 2016). This classification underscores the urgency of stringent control measures to minimize OTA contamination throughout the agricultural and food supply chains.

The molecular formula of OTA is C_20_H_18_ClNO_6_, which is a derivative of phenylalanine and isocoumarin formed by amide linkage. It has stable chemical properties and strong tolerance to adverse environments. Therefore, solving OTA pollution is one of the difficulties in food safety research. Currently, the research on OTA detoxification at home and abroad can be classified into three main categories: physical, chemical, and biological detoxification methods (Ding et al., 2023). Physical methods mainly use adsorption, irradiation and other means to achieve the effect of reducing or detoxification (Yang et al., 2020; Calado et al., 2018). However, physical prevention methods require substantial labor and some techniques can be costly. Chemical methods primarily use chemical substances such as ozone, hydrogen peroxide, sodium hydroxide, and aminomethane to modify the toxin, to make it low-toxic or non-toxic substances (Qi et al., 2016). Long-term use of chemical agents will reduce the control effect, and pollute agricultural products, air, soil and water, endangering human and animal safety and ecological environment. Therefore, finding safe and effective detoxification methods is crucial for resolving OTA contamination issues. At present, biological detoxification is receiving increasing attention. Biological detoxification refers to the degradation of toxins or modification of toxin molecules through biological metabolism or enzyme catalysis to achieve the purpose of detoxification. It has become a current research hotspot because of its high efficiency, low toxicity, strong specificity, low pollution, and no damage to nutrients (Mwabulili et al., 2023).

In recent years, a variety of biocontrol strains for the degradation of OTA have been reported. Probiotics such as Bacillus, lactic acid bacteria, and *Pediococcus pentosaceus* have shown promising results in OTA degradation (Mateo et al., 2022; Qing et al., 2021; Park et al., 2022). Currently, biological control of OTA primarily operates through two forms: bio-adsorption and bio-degradation. Bio-adsorption detoxification refers to the formation of adsorption complexes. Bio-degradation refers to the interaction between microorganisms and their metabolic products with mycotoxins, converting the toxins into less toxic compounds through enzyme-catalyzed reactions. Jahan et al. (2023) isolated a Nudix hydrolase enzyme, Nh-9, from a novel *Bacillus subtilis IS-6* strain using transcriptomic techniques, which efficiently degrades OTA in the matrix. Bacillus subxes between microbial cells or products and toxins, followed by the removal of toxins through filtration or other means. Research has shown that *Lactobacillus plantarum*, *Lactobacillus brevis*, and *Lactobacillus sanfranciscensis* can reduce the concentration of OTA by binding OTA to adsorption (Piotrowska, 2014). The adsorption of mycotoxins by microorganisms is related to the characteristics of the cell wall. The adsorption is not only facilitated by the hydrophobicity of the cell wall but also by the electron donor-acceptor and Lewis acid–base interactions (Piotrowska, 2014). However, mycotoxin adsorption is reversible. The adsorbed toxin can be released under certain conditions, regaining its toxic effect. Therefore, bio-degradation is a more secure apprtilis and Lysobacter have also been found to degrade OTA by producing carboxypeptidases (Qing et al., 2021; Wei et al., 2020). Additionally, amidohydrolases and lipases can also act as OTA-degrading enzymes (Sun et al., 2023). Besides bio-degradation and bio-detoxification, another effective strategy for mycotoxin control involves inhibiting the growth of toxin-producing molds. This can be achieved by methods that compete with the toxigenic molds for nutrients and space or alter their structural integrity, thereby reducing toxin production (Shi et al., 2013; Yu et al., 2021). Despite the extensive research conducted on biocontrol strains for OTA, it remains crucial to expand the library of OTA-degrading strains and to discover novel degrading enzymes. This continuous exploration is essential to develop more effective strategies to mitigate the risks posed by OTA.

Bacillus has many valuable traits and good biocontrol functions. In the past half century, hundreds of bioactive secondary metabolites have been isolated from Bacillus, which make Bacillus have strong application potential in many fields (Aliasghar et al., 2013; Marković et al., 2023). *Bacillus velezensis* is a new species of Bacillus, which is often isolated from marine and river sediments, soil, plant rhizosphere and plant tissues. Because of its wide antibacterial spectrum, rapid growth, easy isolation and culture, strong resistance and high biosafety, it has been widely studied as a probiotic in agriculture, food, industry, medicine and other aspects, and has broad application prospects (Li et al., 2022; He et al., 2022). Studies have shown that *Bacillus velezensis* has strong antagonistic activity against a variety of plant pathogens and can exhibit antifungal activity against plant pathogens by producing a variety of secondary metabolites (Abolfazl et al., 2024). In recent years, studies have found that *Bacillus velezensis* is a probiotic strain with mycotoxin degradation ability. *Bacillus velezensis* has been found to produce lipoproteins and antimicrobial peptides to inhibit the growth of Aspergillus flavus and its spores, showing a good biological control effect on mycotoxins (Wang et al., 2024). At the same time, *B. velezensis* has a good inhibitory effect on some nematodes and insects (Hu et al., 2022). Therefore, *B. velezensis* may be a promising candidate for mycotoxin detoxification and bioremediation in food and feed matrices. It can be used in the field of mildew and detoxification of feed raw materials and mildew and detoxification in livestock and poultry breeding. It is a dominant strain for microbial degradation of mycotoxins.

This study screened OTA-detoxification strain from the soil where corn is planted in Luoyang. And the classification of this strain was determined through morphological identification and 16S rRNA molecular identification. The biological characteristics of the strain were explored and verified through genomic analysis, combined with infection experiments, enzyme production experiments, and antibacterial experiments. Finally, the way in which the strain degraded OTA was studied by high performance liquid chromatography (HPLC). This study aims to provide new ideas for the biological detoxification of OTA and lay a foundation for the subsequent practical application of the strain in the future.

## Materials and methods

### Bacterial strain, culture media, and chemicals

*Bacillus velezensis* MM35 was obtained from a corn planting soil in Luoyang and preserved in the laboratory of livestock and poultry health and functional microbiology of Henan University of Science and Technology with glycerol at −80°C. *B. velezensis* MM35 grew on LB medium (yeast powder 5 g/L, NaCl 10 g/L, tryptone 10 g/L, agar 15 g/L) at 37°C. OTA standard was purchased from Pribolab Biological Engineering (Qingdao) Co., Ltd.; OTA ELISA detection kit and OTA immunoaffinity column were purchased from HUAAN MAGNECH Biotechnology Co., Ltd.; High Performance Liquid Chromatograph grade solvent was purchased from Tianjin Kemiou Chemical Reagent Co., Ltd.; High Performance Liquid Chromatograph purchased from Waters.

### Screening of OTA-degrading strains

The collected soil, animal manure, moldy feed and other samples were taken 1 g each, added with 9 mL sterile water, fully shaken and mixed, and allowed to stand for 30 min to prepare a suspension. The suspension was diluted by 10 times series. The diluted suspension was evenly coated on LB solid medium containing OTA (0.4 μg/mL), and cultured in an incubator at 37°C for 24 h. The obtained strains were inoculated in LB liquid medium containing OTA (0.4 μg/mL) and cultured under 37°C for 48 h. The OTA residue was detected according to the instructions of the OTA ELISA kit and the degradation rate was calculated. The strain with the highest degradation rate was selected for subsequent experiments.

### Morphological identification and molecular identification of OTA-degrading strain

The purified degrading bacteria were streaked on LB medium and cultured overnight in a 37°C incubator to observe the colony morphology. Single colonies were picked for Gram staining, and the morphology of the bacteria was observed under an optical microscope.

In order to further identify the strain, the 16S rRNA method was used to analyze the gene sequence of the strain. The 16S rRNA of the strain was identified by PCR using the DNA of the degrading bacteria as a template [universal primer, 27F (5′-GAGTTTGATCCTGG CTCAG-3′); 1492R (5′-GTTACCTTGTTACGACT-3′)]. Nucleic acid electrophoresis was performed with 1% agarose gel and observed by gel imaging system. The PCR products were sent to Sangon Biotech (Shanghai) Co., Ltd. for sequencing. The sequencing sequence was analyzed by BLAST, and the target sequence was compared with the sequence with high homology by Neighbor-Joining method in MEGA 7.0 software package, and the phylogenetic tree was constructed.

### Whole genome sequencing, prediction and analysis of OTA-degrading strain

The genome DNA of the degrading bacteria was extracted using a bacterial genomic DNA extraction kit. The whole genome sequencing of the strain was performed by Sangon Biotech Co., Ltd. (Shanghai, China).

### Genome sequencing and splicing of OTA-degrading strain

The quality of the raw data was evaluated by FastQC. The quality of Illumina sequencing data was cut by Trimmomatic to obtain relatively accurate and effective data. The second-generation sequencing data were spliced using SPAdes. GapFiller is used to repair the gaps in the contigs obtained by splicing. PrInSeS-G is used for sequence correction to correct editing errors and insertions and deletions of small fragments in the splicing process.

### Functional annotation of OTA-degrading strain genome

The genome characteristics of the strain were analyzed according to the sequencing results, and the genome circle map was drawn by ChiPlot^1^ (visit time was July 23, 2024). Functional annotation of genome sequences was performed with a variety of annotation tools. Firstly, by comparing with the NR library, the similarity of the gene sequence of this strain with similar species was checked.^2^ NCBI Blast+ was used to compare the gene protein sequence with the COG database to obtain its functional annotation information.^3^ GO function annotation information was obtained according to the annotation results of genes and Swissprot and TrEMBL (see footnote 2). KEGG annotation information was obtained by KAAS (see footnote 2).

### Carbohydrate active enzyme annotation of OTA-degrading strain

The carbohydrate active enzyme database (CAZymes^4^) is a professional database related to carbohydrate active enzymes, including related enzyme families that can catalyze carbohydrate degradation, modification, and biosynthesis. HMMER3 was used to compare the gene set protein sequence with the CAZy database to obtain the corresponding carbohydrate active enzyme annotation information.

### Secondary metabolite analysis of OTA-degrading strain

The obtained genome sequence of the degrading bacteria was compared with the secondary metabolite database, and the type and function of the secondary metabolites of the degrading bacteria were analyzed at the genetic level. AntiSMASH (Antibiotics and Secondary Metabolite Analysis Shell) online annotation site^5^ was used to identify secondary metabolite gene clusters.

### Prediction of OTA-degrading enzymes

The reported enzyme sequences were downloaded from NCBI, and the similar sequences were screened in the whole genome of OTA-degrading strain by TBtools 1.115 software. Then the protein structure with high similarity was predicted.

The online website NPS @: SOPMA secondary structure prediction-NPSA^6^ was used to predict the protein secondary structure. The online website SWISS-MODEL^7^ was used to predict the protein tertiary structure.

### Biological characteristics of OTA-degrading strain

#### Stress resistance of OTA-degrading strain

The degrading bacteria were inoculated in LB liquid medium, the survival of strains under different conditions was detected by plate colony counting method. Each group had three replicates. The acid resistance and bile salt resistance of the OTA degrading strain were tested, and the high temperature resistance of the strain was detected.

#### Enzyme-producing test of OTA-degrading strain

The OTA-degrading strain were mixed with 100 μL of 0.5% agar normal saline, and 10 μL of uniform bacterial precipitation was added dropwise to different enzyme-producing media and cultured in a 37°C incubator for 24 h. Observe whether there is a hydrolysis circle around the colony in each enzyme-producing medium, and analyze the enzyme-producing characteristics of the strain through the hydrolysis circle.

#### Bacteriostatic test of OTA-degrading strain

The bacteriostatic experiment was carried out with *Escherichia coli* ATCC25922, *Salmonella typhimurium* ATCC14028 and *Staphylococcus aureus* ATCC6538 as indicator bacteria. Each group had 3 replicates. Observe for the presence of inhibitory circles after 12 h of incubation. The antibacterial activity of the supernatant of OTA-degrading strain against the indicator bacteria was determined according to the diameter of the inhibition zone.

#### Detection of surface hydrophobicity, self-agglomeration ability and co-agglomeration ability of OTA-degrading strain

A single degrading bacterial colony was picked to LB liquid medium, cultured overnight and centrifuged at 8,000 r/min for 10 min at 4°C to collect the bacteria. The bacteria were washed with PBS and resuspended, and the bacterial concentration was adjusted to OD600 = 0.6 ± 0.05 (A_0_). Add 3 mL bacterial liquid and 1 mL xylene vortex to the test tube for 30 s, then stand at 37°C for 1 h, and detect the OD600 (A) of the bacterial liquid by spectrophotometer. At the same time, 2 mL bacterial solution was vortexed for 10 s and allowed to stand at 37°C for 2 h. The supernatant was taken to determine OD600 (A_t_). Two milliliter of bacterial solution was mixed with 2 mL of pathogenic bacteria (OD600 = 0.6 ± 0.05, A_x_), vortexed for 30 s, and placed at 37°C for 4 h to detect OD600 (A_0 + x_). Each group had three replicates.


$$\begin{array}{l} \mathrm{Hydrophobicity}\;\left(\%\right)=[(A_{0}A/A_{0}]\times 100;\mathrm{self}\\ \;-\mathrm{agglomeration rate}\;\left(\%\right)=\left[\left(A_{0}-A_{t}\right)/A0\right]\\ \times 100;co-\mathrm{agglomeration rate}\;\left(\%\right)\\ \;=\left[1–2\times A_{0+x}/\left(A_{0}+A_{x}\right)\right]\times 100. \end{array}$$

#### Infection experiment of OTA-degrading strain on IPEC-J2 cells

The well-grown IPEC-J2 cells were inoculated into 24-well cell culture plates at a density of 2 × 10^5^ cells / well and cultured overnight at 37°C with 5% CO_2_ to obtain monolayer cells. 1.5 h before bacterial inoculation, the cell culture medium was replaced with DMEM medium containing 10% FBS and without antibiotics. The OTA-degrading strain in logarithmic growth phase was added to the cells at a MOI ratio of 100: 1. The cells were collected at different time points after infection to detect the adhesion, invasion and intracellular proliferation of OTA-degrading strain on IPEC-J2 cells. Three replicates were set for each group.

#### The OTA degradation characteristics of MM35 were detected by HPLC

The purified degrading bacteria were cultured in LB liquid medium at 37°C for 48 h, and centrifuged at 5,000 r/min for 10 min at 4°C. The culture supernatant and bacteria were separated, and the bacteria were resuspended with PBS to obtain the bacterial suspension. The 30 mL bacterial suspension was ultrasonically broken to extract the intracellular crude protein. OTA was added to the culture supernatant, intracellular crude protein, bacterial suspension and the same volume of PBS of the degrading bacteria, so that the final concentration was 0.5 μg/mL. Oscillation at 37°C for 48 h, and three replicates were set in each group. According to the national food safety standard GB5009.96-2016, the OTA degradation activity of each component of the degrading bacteria was detected by HPLC.

In order to further explore OTA degradation mechanism of by MM35, we further analyzed the effective components. The effective components of the degrading bacteria were treated with proteinase K (1 mg/mL, 37°C for 1 h), proteinase K + SDS (1 mg/ml proteinase K + 1% SDS, 37°C for 6 h) and heat treatment (boiling water bath for 10 min), respectively. OTA was added to the above components to a final concentration of 0.5 μg/mL, and three replicates were set in each group. The above treatment solution was filtered with a 0.2 μM filter and the OTA residue in each group was detected by HPLC.

Chromatographic conditions: C18 column (5 μm 4.6 × 150 mm); mobile phase: acetonitrile: water: glacial acetic acid = 48: 51: 1; flow rate: 1.0 mL/min; column temperature: 35°C; injection volume: 50 μL; detection wavelength: excitation wavelength 333 nm, emission wavelength: 460 nm.

#### Degradation of OTA in different feed materials by MM35

Corn flour, soybean meal powder, and bran were crushed through a 40-mesh sieve, and the feed raw materials passed through the sieve were sterilized. Under sterile conditions, water (containing OTA standard) was added to the feed sample for contamination, and the sample was fully stirred to evenly mix the feed raw material with the OTA standard. The initial moisture content was 40%, and the final content of OTA in the sample was 150 μg/kg. The prepared contaminated corn flour, soybean meal and wheat bran samples were weighed and 50 g each in the sterilized fermentation flask. The fermentation broth of MM35 strain was inoculated into each contaminated sample at 10% inoculation amount, and the blank control group was replaced with the same amount of sterile water. Each group had 3 replicates, and the fermentation was carried out in a 37°C incubator for 48 h. After the culture, the samples were taken out and dried to detect the residual amount of OTA in different feed ingredients.

### Statistical analysis

Each experiment was repeated three times independently. SPSS 22.0 software was used to analyze the data and GraphPad Prism was used to draw the chart. The results were expressed as mean ± standard deviation (X ± SD). *p* > 0.05 indicated no significant difference, 0.01 < *p* ≤ 0.05 indicated significant difference, *p* ≤ 0.01 indicated extremely significant difference.

## Results

### Screening of OTA-degrading strains

After preliminary isolation and screening of soil, moldy feed, animal manure and other samples collected from different regions, a total of 57 microbial strains with potential degradation of OTA were obtained. The purified strain was inoculated in LB liquid medium containing OTA (0.4 μg / mL) standard, and cultured at 37°C and 200 r/min for 48 h. The OTA residue was detected by ELISA kit. Among them, there were 8 strains with OTA degradation rate of more than 15%, and the strain numbers were MM43, MM15, MM12, MM28, MM39, MM25, MM9, and MM35, respectively. Among them, the strain MM35 has the highest degradation efficiency of OTA, up to 87.10%, so which was selected for follow-up experiments in this study.

### Identification of OTA-degrading strain

The colony of strain MM35 on solid LB medium is white, irregularly round, with wrinkles on the surface. Gram staining microscopy showed blue-purple and uniform color. In addition, the bacteria were rod-shaped, spore-forming and Gram-positive bacillus by optical microscope.

The results of the 16S rRNA gene sequencing for the MM35 strain revealed a specific target band of approximately 1,500 bp using PCR amplification. The PCR product was sequenced and the sequence length of the strain MM35 was 1,452 bp. The sequence was submitted to GenBank and the accession number was ON202821.

**Figure 1:**
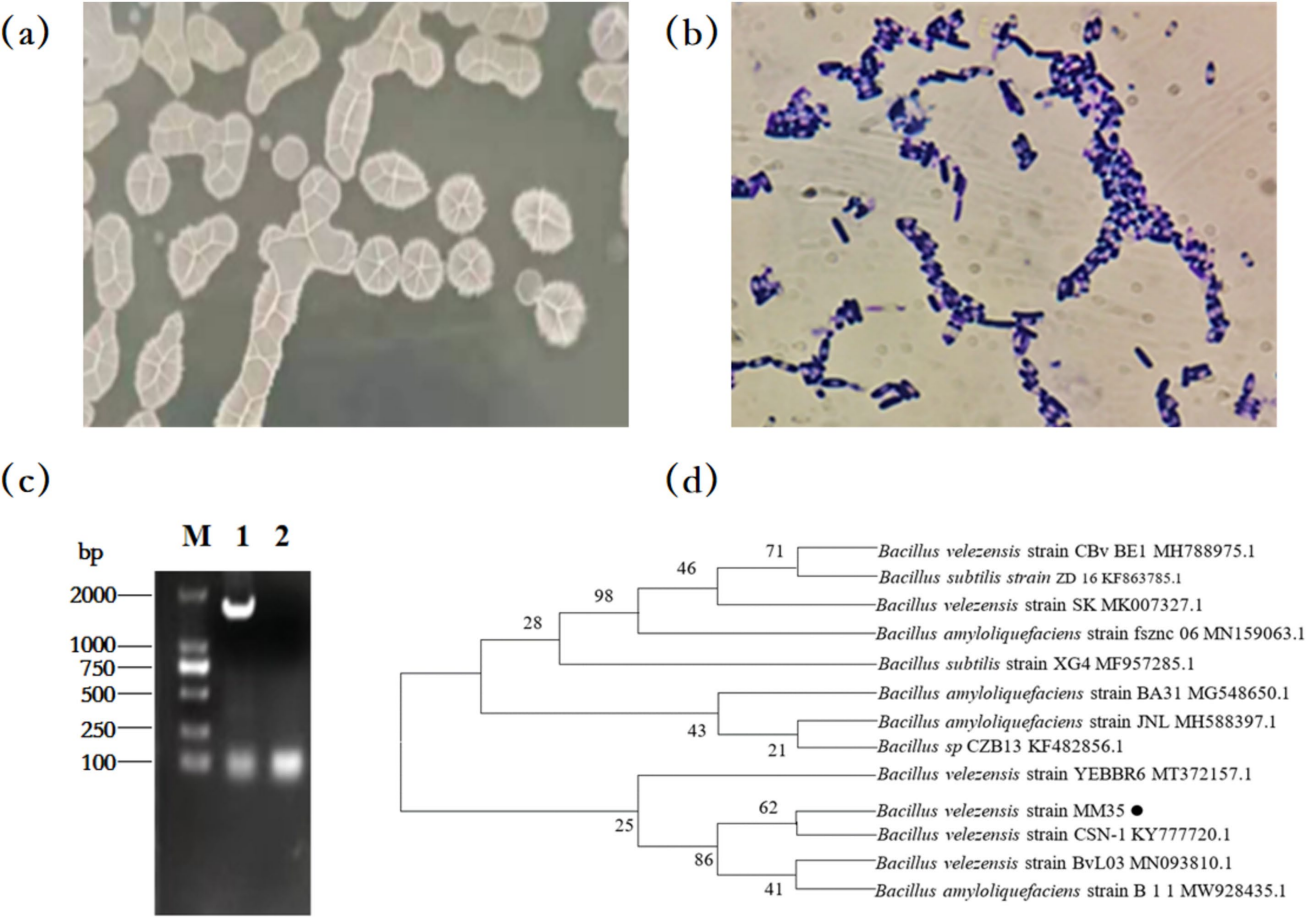
Morphological characteristics and phylogenetic tree of OTA-degrading MM35 strain. **(A)** Cultures of the MM35 strain cultivated on LB agar medium for 12 h at 37°C. **(B)** Gram staining of the MM35 strain observed through a light microscope (magnification 40×). **(C)** Results of 16S rRNA PCR amplification of strain MM35:M: Marker DL 2000; 1:MM35 PCR product; 2: Negative control. **(D)** Phylogenetic tree was constructed based on 16S rRNA gene sequencing results.

The sequencing results were compared by BLAST to construct a phylogenetic tree. Strain MM35 and *Bacillus velezensis strain* CSN-1 KY7777 20.1 were in the same branch, and the genetic relationship was the closest. Therefore, the strain MM35 was identified as *Bacillus velezensis* (Figure 1).

### Genome-wide analysis of OTA-degrading strain

#### Genome characteristics analysis

**Figure 2:**
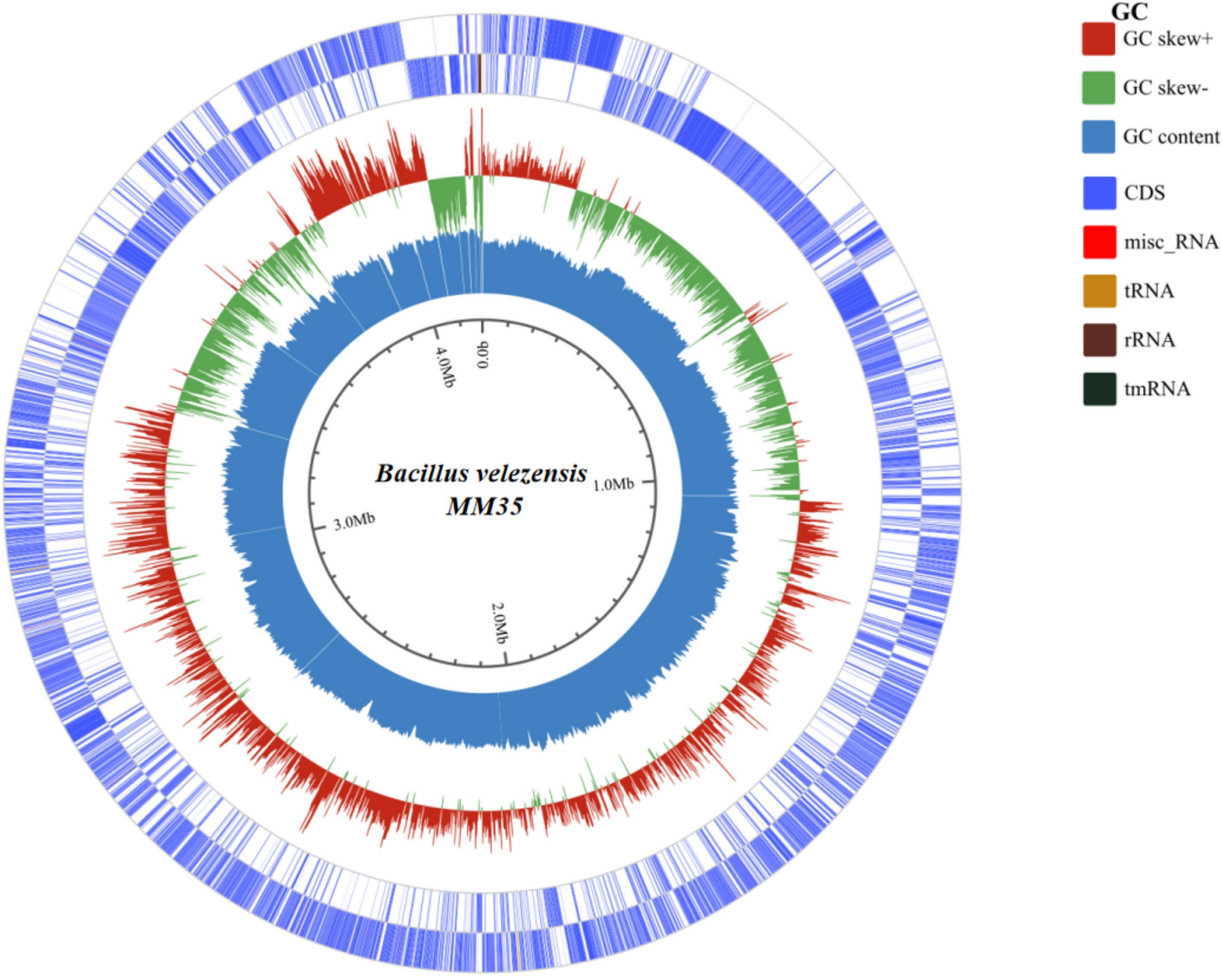
The whole genome circle map of *Bacillus velezensis* MM35. The circles are labeled from the outside to the inside: the first and second circles symbolize the forward and reverse chains of genes associated with CDS, tRNA, and rRNA. The third circle symbolizes the deviation in GC control. The fourth circle represents the GC percentage of the genome.

The genetic features of *B. velezensis* MM35 was displayed in Figure 2 and Table 1. The total base number of *B. velezensis* MM35 was 4.19 Mb, the total genome length was 3,745,398 bp, and the GC content was 46.03%. The genome of the strain contained 4,331 coding genes, including 81 tRNAs and 9 rRNAs. The average length of the coding genes was 864.79 bp.

**Table 1:** General genome features analysis of *Bacillus velezensis* MM35.

Class	Number
Size (base)	4,186,709
Total coding gene (base)	3,745,398
G + C content (%)	46.03%
Protein coding genes	4,331
Average length (base)	864.79
Coding ratio (%)	89.46%
tRNA	81
rRNA	9

#### NR database analysis of *Bacillus velezensis* MM35

**Figure 3:**
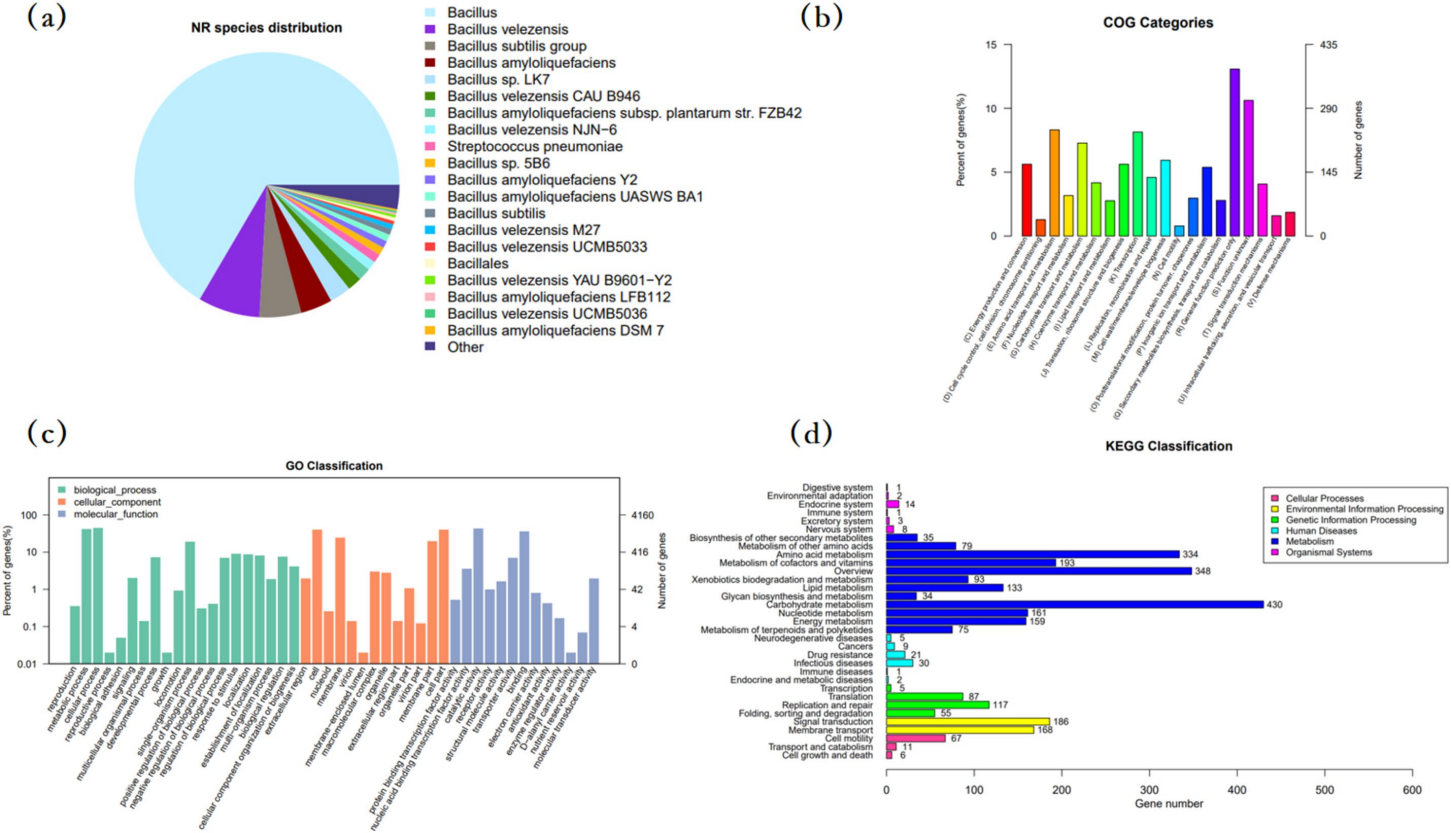
Functional annotation of *Bacillus velezensis* MM35 genome. **(A)** Pie chart of the homology distribution. **(B)** COG functional annotation classification map. **(C)** GO annotation classification map. **(D)** Taxonomic map annotation of the genomic KEGG metabolic pathway.

The sequence of MM35 strain was compared with NR database, and the pie chart of MM35 homology distribution was drawn. As shown in Figure 3, strain MM35 has high homology with Bacillus and *Bacillus velezensis*.

#### COG functional annotation of *Bacillus velezensis* MM35

In order to annotate the protein function of *B. velezensis* MM35, the COG database was selected for annotation analysis of its genome. The number of proteins annotated in the COG database of the *B. velezensis* MM35 was 2,900, of which R predicted that there were up to 379 proteins performing basic functions. Followed by E amino acid transport metabolism, K transcription and G carbohydrate transport and metabolism, respectively, 241,236 and 211 (Table 2).

**Table 2:** COG categories of *Bacillus velezensis* MM35.

Code	Name	Gene num	Gene ratio
C	Energy production and conversion	163	5.62
D	Cell cycle control, cell division, chromosome partitioning	37	1.28
E	Amino acid transport and metabolism	241	8.31
F	Nucleotide transport and metabolism	92	3.17
G	Carbohydrate transport and metabolism	211	7.28
H	Coenzyme transport and metabolism	121	4.17
I	Lipid transport and metabolism	80	2.76
J	Translation, ribosomal structure and biogenesis	163	5.62
K	Transcription	236	8.14
L	Replication, recombination and repair	133	4.59
M	Cell wall/membrane/envelope biogenesis	172	5.93
N	Cell motility	23	0.79
O	Posttranslational modification, protein turnover, chaperones	86	2.97
P	Inorganic ion transport and metabolism	156	5.38

#### GO functional annotation of *Bacillus velezensis* MM35

GO functional annotation provides a comprehensive description of the properties of genes and gene products in an organism. In the biological process classification of GO functional annotation of *B. velezensis* MM35, the number of genes annotated to participate in cellular processes and metabolic processes was the largest, 1,835 and 1751, respectively. In the classification of cell components, the genes involved in cells and cell components were the most, all of which were 1,669. In the molecular function classification, the number of genes involved in catalytic reaction and binding was the highest, which were 1,765 and 1,476, respectively. A large number of genes in *B. velezensis* MM35 are responsible for cellular processes, metabolic processes and catalytic activity, cell and binding. On the whole, the number of genes involved in bacterial cell process and catalytic activity in *B. velezensis* MM35 is the largest.

#### KEGG functional annotation of *Bacillus velezensis* MM35

KEGG is a comprehensive database of biological systems with integrated bases information on group, chemical and system functions. In the KEGG functional annotation classification of *B. velezensis* MM35, the number of genes involved in carbohydrate metabolism, amino acid metabolism and metabolic pathways involved in cofactors and vitamins was the largest, with 430, 334, and 193, respectively. The number of genes related to signal transduction, membrane transport, nucleotide metabolism and energy metabolism was second.

#### Carbohydrate active enzyme annotation of *Bacillus velezensis* MM35

**Figure 4:**
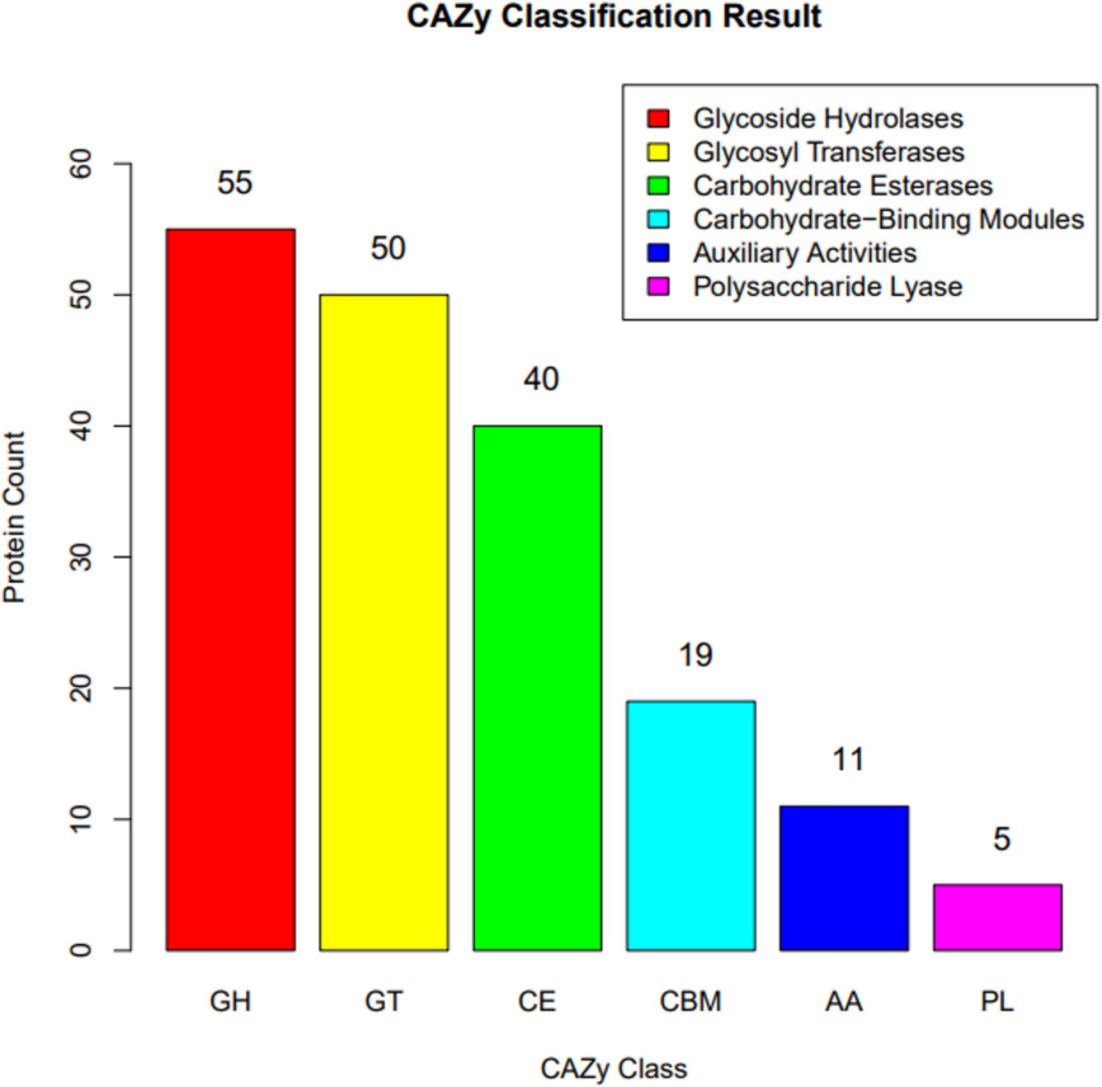
Distribution of the carbohydrate active enzyme (CAZy) family protein identified in the genome of *Bacillus velezensis* MM35.

The genomic sequence analysis of *B. velezensis* MM35 identified 180 CAZymes family enzymes, including 55 GHs, 50 GTs, 40 CEs, 19 CBMs, 11 AAs, and 5 PLs. Among them, the number of genes encoding glycoside hydrolase was the largest, followed by glycosyltransferase and carbohydrate esterase, accounting for 30.6, 27.8, and 22.2% of the total number of CAZymes genes, respectively (Figure 4).

#### Analysis of secondary metabolites of *Bacillus velezensis* MM35

**Figure 5:**
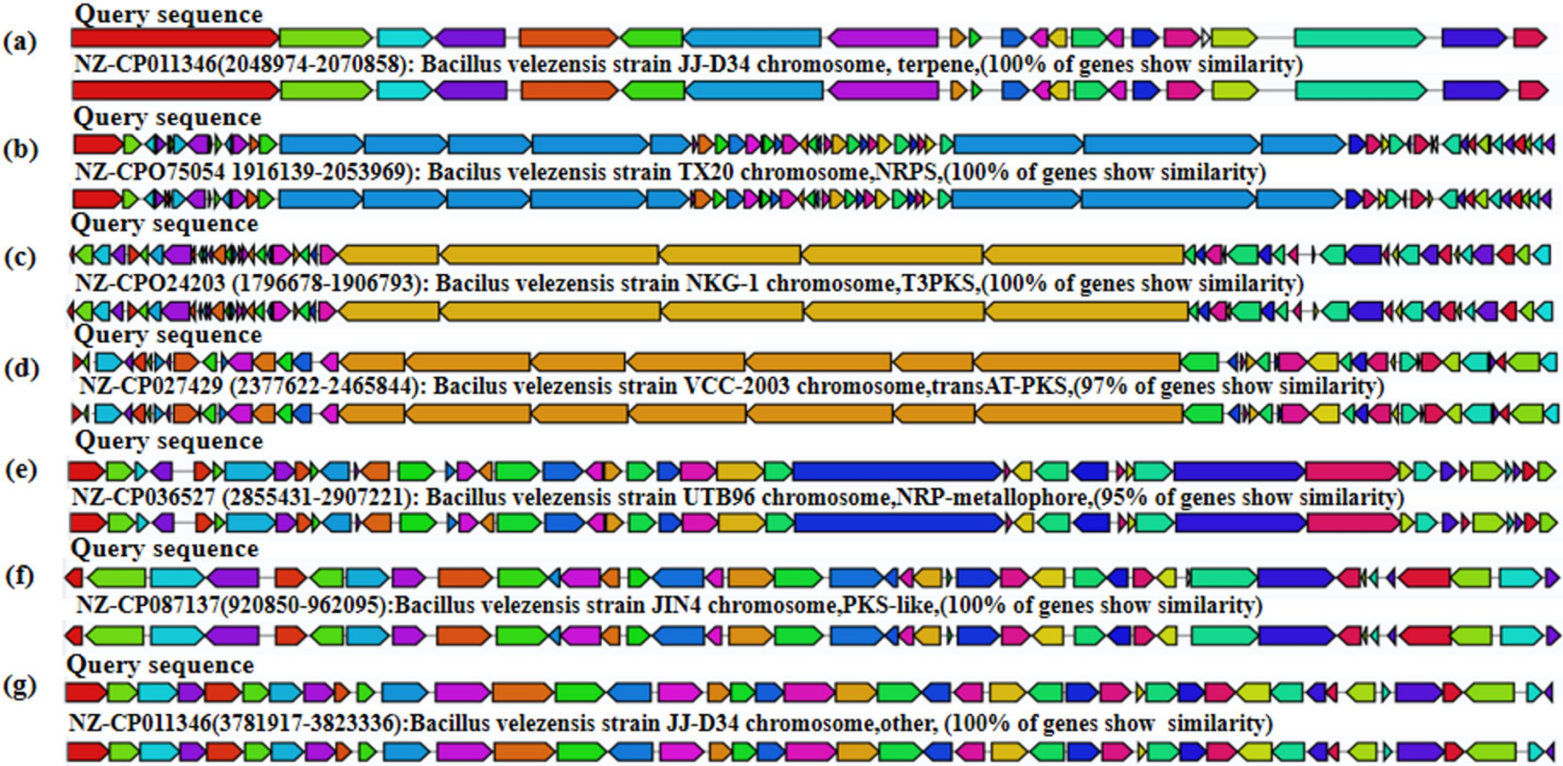
Analysis of secondary metabolites of *Bacillus velezensis* MM35. **(A)** Terpene. **(B)** NRPS. **(C)** T3PKS. **(D)** transAT-PKS. **(E)** NRP-metallophore. **(F)** PKS-like. **(G)** Other.

The obtained *B. velezensis* MM35 genome sequence was compared with the secondary metabolite database, and a variety of secondary metabolites encoding antibacterial properties were identified in *B. velezensis* MM35. The *B. velezensis* MM35 encodes 15 gene clusters related to secondary metabolites, of which 7 are completely consistent or highly similar to the identified biosynthetic gene clusters. The whole genome of *B. velezensis* MM35 contains four gene clusters encoding NRPS (non-ribosomal peptide synthase), four gene clusters encoding transAT-PKS (trans-AT-type polyketide synthase), two gene clusters encoding terpene, and multiple gene clusters encoding T3PKS (Type III Polyketide Synthases) and LAP (Figure 5).

#### Structure prediction of OTA degrading enzyme in *Bacillus velezensis* MM35

**Figure 6:**
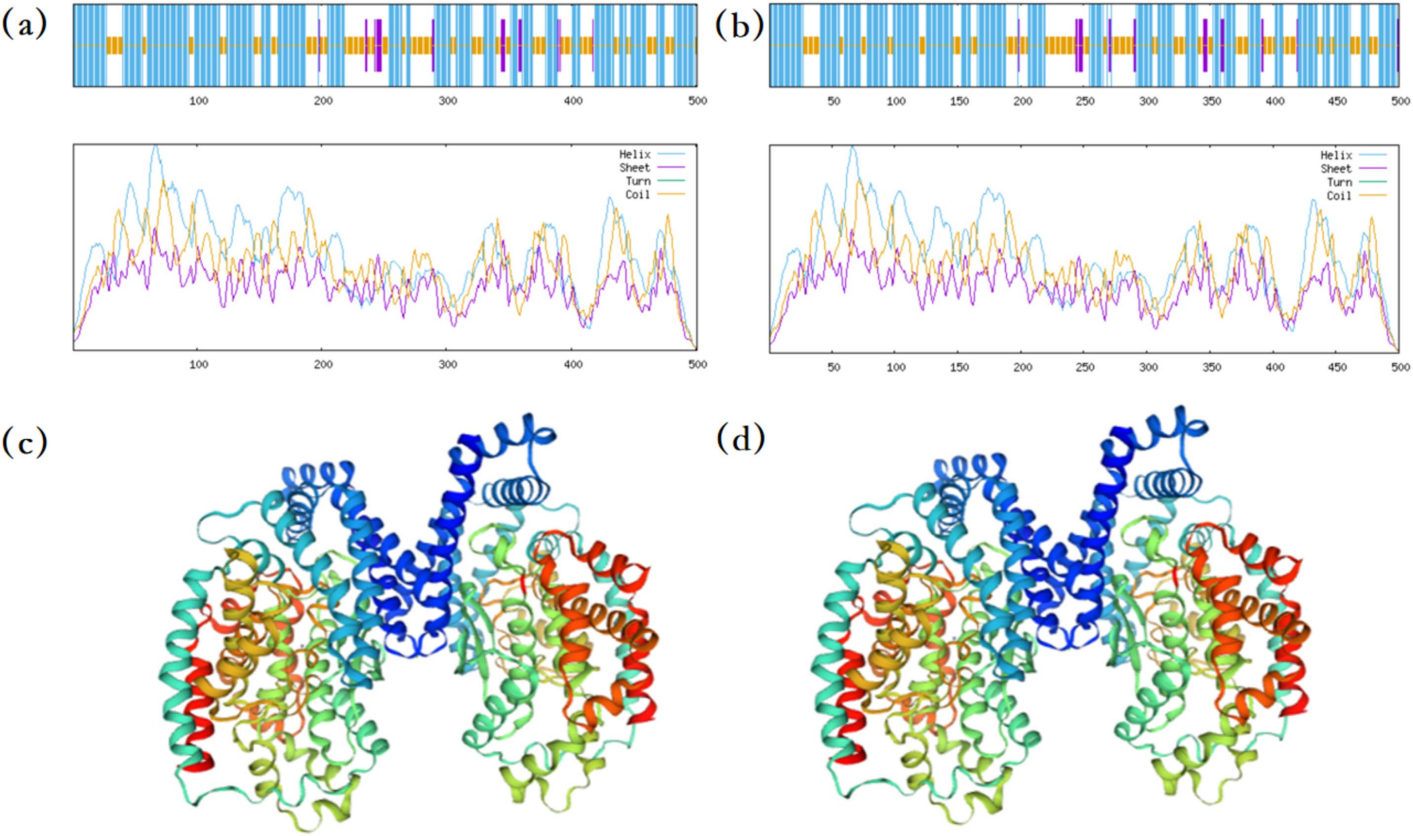
Prediction of protein structure. **(A)** The secondary structure of carboxypeptidase has been reported for prediction. **(B)** Prediction of secondary structure of carboxypeptidase in MM35 strain. **(C)** The tertiary structure of carboxypeptidase has been reported for prediction. **(D)** Prediction of tertiary structure of carboxypeptidase in MM35 strain.

The protein sequence of *B. velezensis* MM35 was compared with various known protein sequences such as carboxypeptidase, amidase, laccase and lactone hydrolase. The results showed that the protein sequence numbered PROKKA_03629 in *B. velezensis* MM35 was as high as 59.3% with a known carboxypeptidase. The protein structure of the two was predicted. In the secondary structure of the carboxypeptidase, *α*-helix accounted for 61.68%, extended chain accounted for 4.19%, and random coil accounted for 34.13%. Similarly, in the secondary structure of PROKKA_03629, α-helix accounted for 60.40%, extended chain accounted for 4.40%, and random coil accounted for 35.20%. In addition, the locations of various secondary structures in the two proteins are highly similar, and the tertiary structures of the two proteins are also very similar (Figure 6).

### Biological characteristics of *Bacillus velezensis* MM35

#### Stress resistance of *Bacillus velezensis* MM35

The survival rate of *B. velezensis* MM35 could reach 80.56, 69.57, and 59.34% after treatment with pH 4.0, 3.0, and 2.0 for 3 h. The survival rate can still reach 82.16, 71.61, and 60.47% after cultured in PBS with bile salt concentration of 0.1, 0.2, and 0.3% for 6 h. In addition, 6 × 10^7^ CFU strain still had a survival of 2.2 × 10^6^ CFU after treatment at 100°C for 30 min. The results show that the strain has certain tolerance to acid and bile salts, and can tolerate high temperature, which is a strain with good stress resistance.

#### Enzyme production results of *Bacillus velezensis* MM35

**Figure 7:**
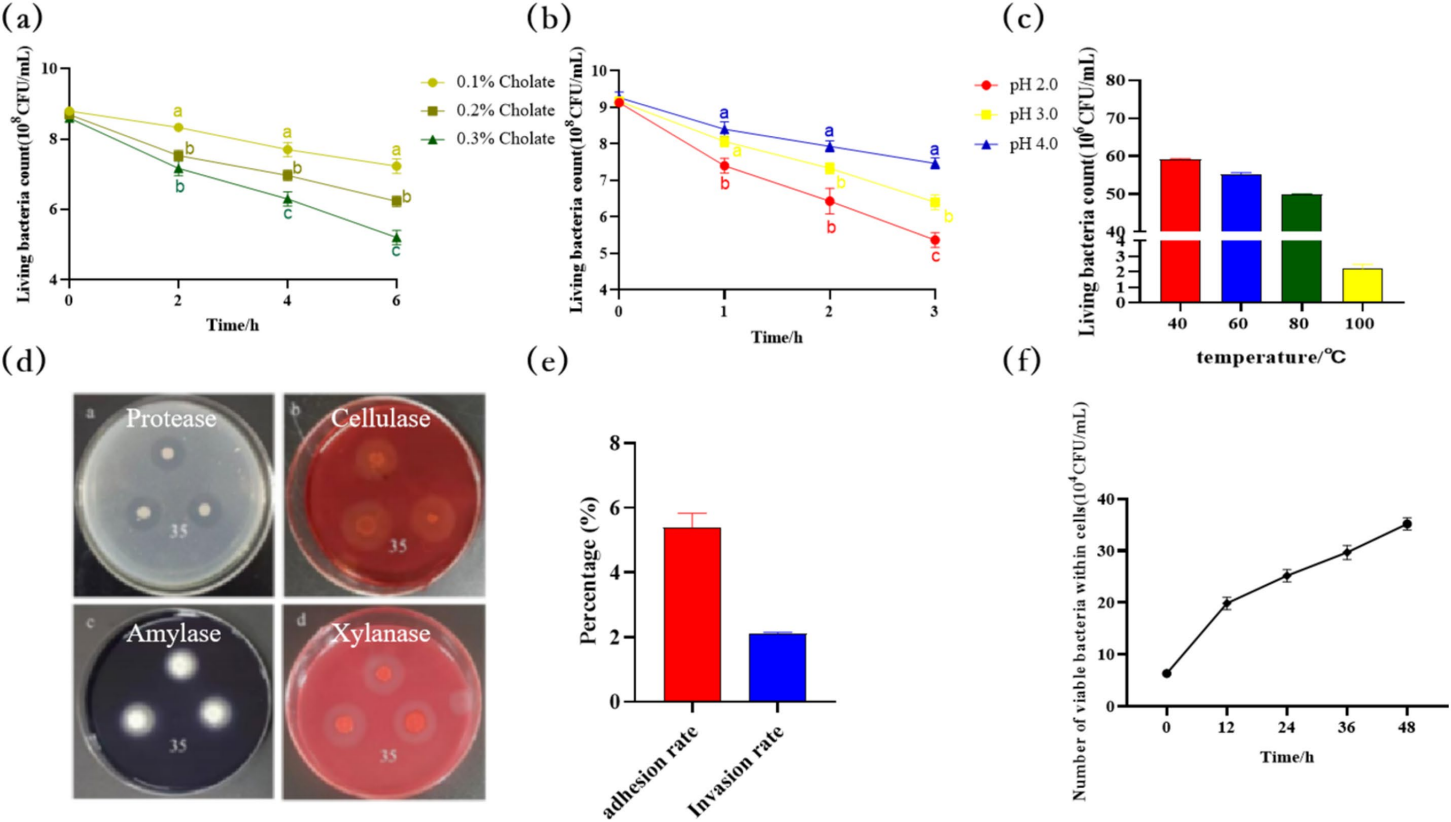
Physiological property of *Bacillus velezensis* MM35. **(A)** The survival of *Bacillus velezensis* MM35 at pH 4.0, 3.0, and 2.0 for 3 h. Different lowercase letters in the figure indicate that there is a statistically significant difference between the treatment groups in this time period (*p* < 0.05). **(B)** The survival of strain MM35 cultured in PBS with bile salt concentration of 0.1, 0.2, and 0.3% for 6 h. Different lowercase letters in the figure indicate that there is a statistically significant difference between the treatment groups in this time period (*p* < 0.05). **(C)** The survival of strain MM35 after treatment at 40, 60, 80, 100°C for 30 h. **(D)** Enzyme production result: a: Protease; b: Cellulase; c: Amylase; d: Xylanase. **(E,F)** Analysis of the colonization ability of *Bacillus velezensis* MM35 on IPEC-J2 cells.

The hydrolysis circles in the medium were observed. As shown in the Figure 7, the *B. velezensis* MM35 showed hydrolysis circles in the protease medium, sodium carboxymethyl cellulose medium supplemented with Congo red staining solution, xylan medium and amylase medium, indicating that the strain could produce protease, cellulase, xylanase and amylase.

#### Antibacterial results of *Bacillus velezensis* MM35

The antibacterial ability of the supernatant of *B. velezensis* MM35 was tested by Oxford cup method. The average antibacterial diameter of *B. velezensis* MM35 against *Escherichia coli* ATCC25922 was 16.62 ± 0.32 mm, the average antibacterial diameter against *Staphylococcus aureus* ATCC6538 was 15.82 ± 0.17 mm, and the average antibacterial diameter against *Salmonella typhimurium* ATCC14028 was 16.33 ± 0.45 mm. The antibacterial effect of *B. velezensis* MM35 on *Escherichia coli* ATCC25922 was slightly higher than that of *Staphylococcus aureus* ATCC6538 and *Salmonella typhimurium* ATCC14028.

#### Surface hydrophobicity, self-agglomeration ability and co-agglomeration ability of *Bacillus velezensis* MM35

The hydrophobicity and self-agglomeration ability of bacteria are considered to be one of the important factors affecting the two-phase reaction, which is related to a variety of adhesion phenomena. The hydrophobic rate of *B. velezensis* MM35 was 17.99%, and the self-agglomeration rate was 71.50%. The co-agglomeration rate of the strain with *E. coli* 1,526 was 38.62%, and the coagulation rate with *Salmonella typhimurium* 1,344 was 43.41%.

#### Infection experiment of *Bacillus velezensis* MM35 on IPEC-J2 cells

The results of adhesion and invasion of IPEC-J2 cells by *B. velezensis* MM35 were shown in Figure 7E. The results showed that the adhesion rate of *B. velezensis* MM35 to IPEC-J2 cells was 5.4%, and the invasion rate was 2.1%. In addition, as shown in Figure 7F, the amount of intracellular bacteria of *B. velezensis* MM35 increased with time within 12, 24, 36 and 48 h after bacteria invasion, reaching 3.5 × 10^5^ CFU at 48 h. The results suggested that the *B. velezensis* MM35 could stably colonize in IPEC-J2 cells and showed significant intracellular proliferation advantages.

#### The OTA degradation characteristics of *Bacillus velezensis* MM35 were detected by HPLC

The culture supernatant of *B. velezensis* MM35 showed strong OTA degradation ability. Compared with bacterial suspension and intracellular crude protein, the culture supernatant had a stronger ability to degrade OTA. After incubation at 37°C for 48 h, the degradation rate of OTA in the culture supernatant was 63.95%, while the degradation rates of bacterial suspension and cell extract were 50.29 and 43.30%, respectively. This indicated that the pathway of OTA degradation by *B. velezensis* MM35 was mainly enzymatic degradation, supplemented by biosorption.

**Figure 8:**
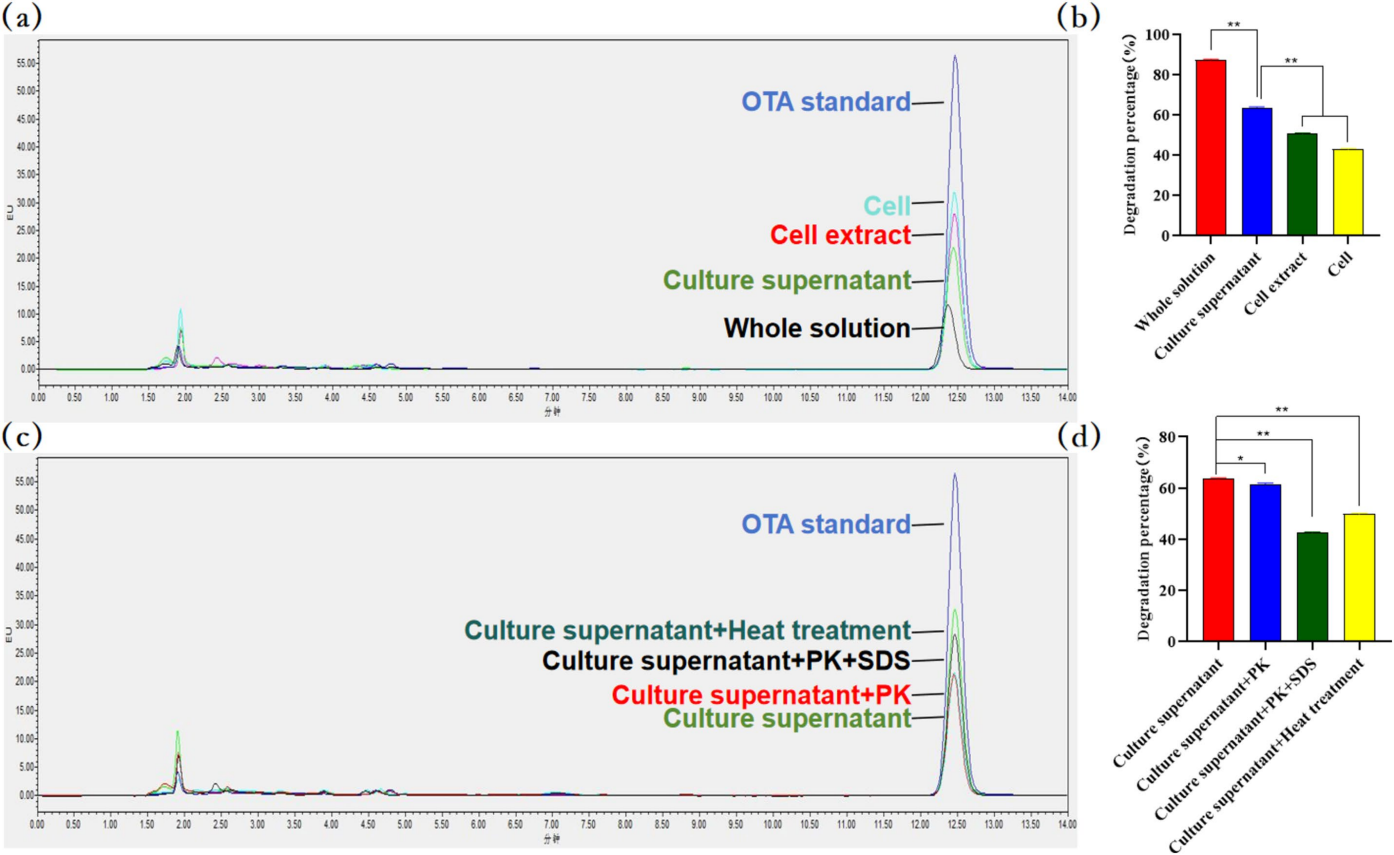
OTA residues were detected by HPLC. **(A,B)** The OTA degradation activity of each component of *Bacillus velezensis* MM35. The supernatant was significantly different from the other two groups (**p < 0.01). **(C,D)** The degradation rate of OTA by *Bacillus velezensis* MM35 culture supernatant after incubation in proteinase K, proteinase K + SDS incubation and heat treatment. After treatment with proteinase K, the degradation rate was significantly lower than that of the culture supernatant (*p < 0.05), and the other two groups were very significantly reduced (**p < 0.01).

Further, we treated the culture supernatant of *B. velezensis* MM35. The results showed that the OTA degradation rate of the culture supernatant after protease k treatment was 61.83%, which was 2.12% lower than that of the culture supernatant. The degradation rate of OTA in the culture supernatant after co-treatment with protease K and SDS was 42.24%, which was 21.71% lower than that in the culture supernatant. In addition, the degradation rate of the culture supernatant after heat treatment was 49.76%, which was 14.19% lower than that of the culture supernatant. This indicates that the extracellular enzyme degrading OTA in the culture supernatant of *B. velezensis* MM35 is not a macromolecular protease, but a small molecular biological enzyme, and the extracellular enzyme has a certain heat resistance (Figure 8).

#### Degradation of OTA in different feed materials by MM35

**Figure 9:**
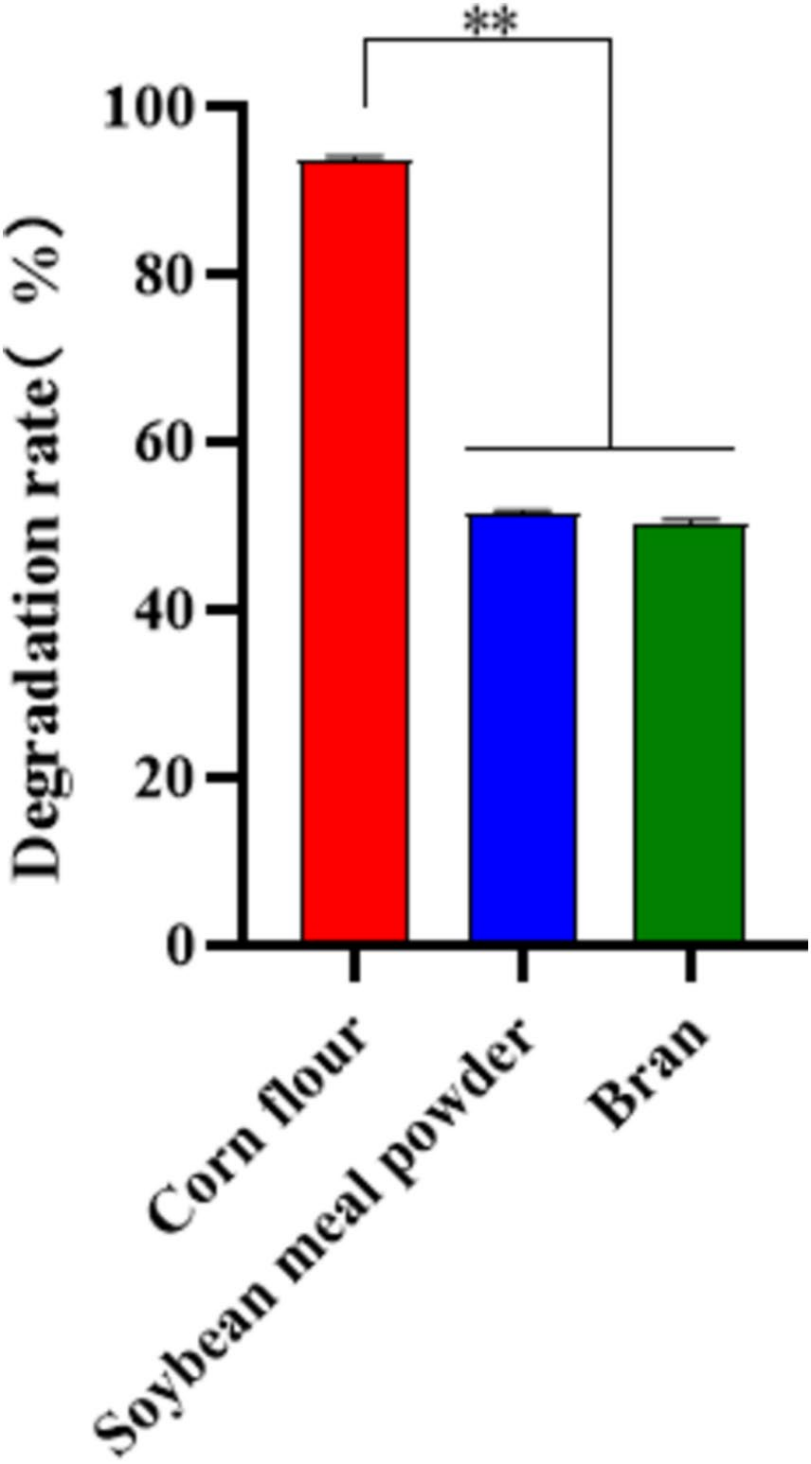
Degradation of OTA in Different Feed Materials by MM35. The degradation rate of OTA in corn flour by MM35 strain was significantly higher than that of the other two groups (**p < 0.01).

Different types of feed ingredients contain different nutrient components, which may lead to different degradation rates of strains in different fermentation substrates. In this study, MM35 had a good degradation effect on OTA in corn flour, and the degradation rate was 93.56%. The degradation effect of OTA in soybean meal and bran was poor, and the degradation rates were 51.41 and 50.16%, respectively (Figure 9).

## Discussion

Due to various environmental influences, regions around the globe experience different levels of mycotoxin contamination. OTA is another mycotoxin that has attracted widespread attention globally after aflatoxins. Improper storage of food or feed can lead to extensive mold growth, resulting in widespread residual OTA in various food and feed products. Prolonged consumption of OTA-contaminated feed by livestock can lead to decreased antioxidant capacity, liver and kidney damage, reduced growth and reproductive performance, and various associated complications (Zhang et al., 2016; Battacone et al., 2010). The consumption of OTA-contaminated feed by animals leads to the accumulation of the toxin in organs, fats, muscle tissue, and blood, ultimately posing risks to human health through the consumption of these contaminated animal products. Its presence severely impacts agricultural production and poses substantial risks to human health (Li et al., 2014). While some physical and chemical detoxification methods have shown certain effectiveness in detoxifying OTA, they may alter the taste of food and feed. In addition, the use of chemical agents can easily cause chemical residues, which seriously affect the health of consumers. Therefore, utilizing probiotics for efficient, uncontaminated biological detoxification of mycotoxins is a current research focus. However, most probiotics have low tolerance, strict requirements for growth and storage conditions, high prices, high production costs, and low application value (Hathi et al., 2021). In this context, the development of novel strains with good stress resistance, ease of production, and excellent clinical application effects for the biological degradation of mycotoxins is of significant practical importance.

In this study, a strain with the ability to degrade OTA was isolated from soil and designated as MM35. Through morphological identification, molecular identification, and comparison with the NR database, the strain was identified as *Bacillus velezensis*. *Bacillus velezensis* is a highly promising probiotic within the Bacillus genus. It is reported that *Bacillus velezensis* can be used as a high-quality feed additive. The extracellular antibacterial substances produced by its fermentation can inhibit the growth of diarrhea-causing pathogens such as *Escherichia coli*, *Salmonella* and *Shigella*, and have good stress resistance. It is an advantageous probiotic with significant potential for development in the field of biofeeds (Khalid et al., 2021).

In recent years, genome sequencing technology has developed rapidly. The whole-genome sequence contains all the genetic information of the strain, which is crucial for discovering gene functions, better understanding the strain’s structure, biological functions, physiological characteristics, and metabolic features (Loman and Pallen, 2015). This study found that *B. velezensis* MM35 annotated 2,900 proteins in the COG database, primarily involved in amino acid transport metabolism, transcription, and carbohydrate transport and metabolism. Additionally, 3,092 genes were annotated in the GO database, mainly related to biological processes, catalytic reactions, and metabolic processes. Furthermore, the *B. velezensis* MM35 was found to be enriched in metabolic-related genes in the KEGG database. These results indicate that the *B. velezensis* MM35 possesses good metabolic and catalytic synthesis capabilities and significant potential for application in carbohydrate and protein synthesis and degradation. In the CAZy annotation results, genes from the GH family were the main annotated genes. The GH family includes families encoding cellulases, such as GH1, GH8, and GH9, as well as genes related to xylanases. Consistent with the predictions, the *B. velezensis* MM35 exhibited characteristics of producing cellulases and xylanases. Moreover, the *B. velezensis* MM35 can also produce proteases and amylases, enhancing the digestibility and absorption of feed in animals, making it an extremely promising probiotic with significant potential for biocontrol.

The antimicrobial activity is considered an important selection criterion for probiotics in animal feed and nutrition (Fusco et al., 2023). Studies have found that *Bacillus velezensis* has strong antifungal activity, which can deform the hyphal structure of fungi, affect biofilm formation, and inhibit collective movement (Zhang et al., 2016). According to the comparison results with the secondary metabolite database, the *B. velezensis* MM35 was identified to contain various secondary metabolites with antimicrobial properties, such as NRPS and terpenoids (Baptista et al., 2022; Gaudelli et al., 2015). Combining the results of the antibacterial experiments, the supernatant of this strain showed inhibitory effects on the growth of pathogenic bacteria such as *Escherichia coli*, *Staphylococcus aureus*, and *Salmonella typhimurium*. Bacterial infection-related diseases pose a significant threat to public health, and inhibiting the growth of pathogenic bacteria on crops is of great significance to production practices. The *B. velezensis* MM35 has strong antibacterial activity and can be further developed into a biological control agent. In addition, the strain showed a high coaggregation ability against pathogenic bacteria such as *Escherichia coli*, indicating its potential to improve the microenvironment of animal intestines and promote their healthy growth and development.

In recent years, probiotics have been widely used in production practices (Mahizan et al., 2019). As a probiotic with OTA-degrading ability, the development of the *B. velezensis* MM35 is inevitably affected by adverse environments such as high temperature and strong acidity. Therefore, exploring the strain’s resistance is of great significance for its subsequent development and research. Results have shown that the *B. velezensis* MM35 can survive in various adverse environments, exhibiting good resistance to high temperature, acidity, and bile salts. This indicates that the strain can not only survive during the high-temperature treatment of feed but also tolerate the acidic environment of the gastrointestinal tract. In addition, the invasion experiment of the strain showed that it has a good adhesion and invasion ability to IPEC-J2 cells and can stably colonize inside the cells. The stable colonization of probiotics in the host’s intestines helps in their reproduction of microbial populations and thus exert their own efficacy.

The enzymatic degradation of OTA mainly involves breaking the molecular bonds of OTA, changing its original molecular structure and properties to achieve detoxification. Its degradation mechanism mainly involves breaking the amide bond to hydrolyze OTA into the non-toxic L-*β*-phenylalanine and OTα (Tae, 2024). As early as 1969, reports indicated that carboxypeptidase A (CPA) could hydrolyze the amide bond to degrade OTA into OTα and L-phenylalanine (Ismaiel et al., 2022). Subsequently, various enzymes with similar functions have been discovered, such as lipase and pancreatic enzymes (Pitout, 1969). In recent years, Wang et al. (2023) isolated a novel degrading enzyme ADH3 from an acidophilic bacterium, and 1.2 μg / mL of ADH3 could completely degrade 50 μg / L of OTA within a short time, converting OTA into the non-toxic OTα through hydrolyzing the amide bond. Hydrolysis of the lactone ring is also a feasible OTA degradation method. Studies have shown that lactone hydrolase can hydrolyze the lactone ring of OTA to produce a low-toxic metabolite OP-OTA (Luo et al., 2022; Leitão and Enguita, 2021). In addition, OTA can also be degraded by hydroxylation to produce ochratoxin hydroquinone (OTHQ) or dechlorination of isocoumarin ring to produce ochratoxin-B (OTB) and further degradation to ochratoxin β (OTβ) (Lu et al., 2022). *Bacillus velezensis* has been repeatedly reported as a biocontrol agent for mycotoxins in recent years (Zhang et al., 2022; Wang et al., 2024). *Bacillus velezensis* can not only inhibit the growth of fungi, but also prevent and control mycotoxins by producing degrading enzymes such as laccase (Li et al., 2020). Wang et al. analyzed 15 mycotoxins in five different types of nuts and dried fruits in China. OTA was found in only two raisin samples with pollution levels of 4.6 and 7.4 μg / kg^−1^, respectively, which were far below the international limit standard (Wang et al., 2018). In addition, studies have shown that the degradation rate of OTA by mycotoxin degrading bacteria is positively correlated with the degradation time (Azam et al., 2019). Wang et al. (2022) found that the degradation efficiency of OTA by *Bacillus amyloliquefaciens* YL-1 could reach 79.7% within 48 h, which could efficiently degrade OTA. In this study, the mechanism of OTA degradation by *B. velezensis* MM35 was preliminarily discussed. In our study, *B. velezensis* MM35 has a good degradation effect on OTA, and the degradation rate of OTA can reach 87.10% within 48 h. In addition, the MM35 strain mainly acts through extracellular enzymes. It is not a macromolecular protease, but a small molecule biological enzyme, which has a certain heat resistance. This may be related to the carboxypeptidase encoded by *B. velezensis* MM35. Further, in this study, OTA in different feed ingredients was degraded. The results showed that MM35 had the highest degradation efficiency of OTA in corn flour (OTA content was 150 μg/ kg), and the degradation rate reached 93.56% within 48 h. This suggests that MM35 strain has great potential in practical application. In addition, as a common probiotic, *Bacillus velezensis* has a variety of antifungal components in its fermentation broth, and its secreted antifungal compounds can significantly inhibit pathogenic fungi (Zhao et al., 2022). Similar to our study, the fermentation supernatant of *B. velezensis* MM35 can degrade OTA while inhibiting the growth of various pathogens. In addition, Marta et al. showed that *B. velezensis* showed high antifungal activity against some plant pathogenic fungi with important economic impact, such as *Fusarium graminearum*, *F. solani*, *F. oxysporum*, and so on (Torres et al., 2020). This further confirms the application potential of *B. velezensis* MM35 in agricultural production.

According to statistics, about 25% of the world’s grains are contaminated by various molds every year. The public health problems caused by mycotoxin contamination have attracted worldwide attention. Researchers have deeply studied the origin, classification, mechanism of action, exposure pathways, health effects and prevention measures of mycotoxins (Abamecha, 2022). Although significant progress has been made, climate change, fungal strain evolution and other factors pose new challenges to the management of mycotoxin contamination (Qian, 2023). Mycotoxins continue to pose a great threat to agriculture, food safety, human and animal health. As an important part of public health, understanding the biochemical complexity of mycotoxins and exploring efficient and safe detoxification methods are of great significance to ensure the safety of the global food chain. Nowadays, the use of probiotics for mycotoxins has attracted great interest (Średnicka et al., 2021; Ndiaye et al., 2022). As a common probiotic, lactic acid bacteria metabolites have high efficiency and strong specificity in inhibiting the growth of Fusarium and removing mycotoxins (Smaoui et al., 2023). It is a promising biological control technology and can be used as preservatives in agricultural management plans and food industries. In addition, zymosan can be used as a detoxification additive for mixed mycotoxins in feed to improve intestinal mucosal integrity and liver metabolic enzyme activity (Zhang et al., 2023). However, in the research process of probiotics such as lactic acid bacteria, there are difficulties such as complicated screening technology, long cycle and low success rate. These conditions restrict the development of these strains in the prevention and control of mycotoxin pollution. Therefore, it is urgent to explore new methods for mycotoxin detoxification. Enzymatic degradation of mycotoxins is considered to be a very friendly method of prevention and treatment. There are abundant microorganisms and new enzyme resources in nature that can be used for the degradation of mycotoxins. How to excavate these undiscovered new enzymes and modify them to meet the production needs has become a research hotspot. With the continuous enrichment of microbial genome data, gene mining of microbial sequences can be more effective and convenient to analyze strain information (Mohd Din and Othman, 2023). Studies have shown that genome mining is a promising method for mining new degrading enzymes. In this study, we combined whole genome sequencing with physiological characteristics to provide more information for exploring the biological function of *B. velezensis* MM35. The results showed that the genomic information of *B. velezensis* MM35 was highly consistent with its physiological characteristics. Genome sequencing analysis may be used as a new method to bring hope for the future development of agriculture, food and other industries.

However, mycotoxin contamination is often manifested as mixed contamination of multiple toxins. Therefore, the detoxification of mycotoxins by single probiotics or biological enzymes cannot completely prevent the contamination of mycotoxins, which has great limitations. The application of microbial consortium provides a promising solution for the effective co-degradation of multiple mycotoxins. Compound probiotics and biological enzyme modification have attracted people’s attention as new research strategies. With the development of genetic engineering technology, recombinant degrading enzymes are becoming more and more popular in biological detoxification. It is necessary to develop recombinant degrading enzymes that can degrade a variety of toxins. However, most biological detoxification technologies have not yet developed to a commercial scale, because the potential health effects of degradation pathways and degradation products have not been thoroughly understood. In order to promote the commercial application of these methods, future research should focus on explaining detailed degradation mechanisms, conducting toxicological studies on metabolites, and developing new analytical methods. In view of the diversity of complex matrices, further research is recommended to highlight *in-situ* degradation techniques suitable for mycotoxin control. In the future, efforts should be made to explore cost-effective, efficient, simple and easy-to-operate mycotoxin biodegradation strategies for large-scale industrial applications.

In summary, *B. velezensis* MM35 is a dominant probiotic that can efficiently degrade OTA. The strain can resist harsh environment and has good stress resistance. Furthermore, the strain also has strong enzyme production ability, antibacterial ability and stable colonization ability in the intestine. These advantages have laid a theoretical foundation for its subsequent development and research. In addition, we excavated a carboxypeptidase with OTA degradation potential from the genome of *B. velezensis* MM35. In the future, we will further study the structure, function and OTA degradation ability of this carboxypeptidase to promote its application in food and feed processing. The discovery of *B. velezensis* MM35 provides a new material for the biodegradation of OTA in feed. *B. velezensis* MM35 can be used as a high-quality feed additive in production practice, so as to promote the development of animal husbandry and ensure food safety.

## Data Availability

The 16s rRNA sequence is available at the NCBI with accession number: ON202821. Illumina sequence is available at the NCBI with accession number: JBJOUE000000000.

## Footnotes

^1^ https://www.chiplot.online/

^2^ http://www.geeontology.org

^3^ https://www.ncbi.nlm.nih.gov/COG/

^4^ http://www.cazy.org/

^5^ http://antismash

^6^ https://npsa.lyon.inserm.fr

^7^ https://swismodel.expasy.org
